# The first South American case of pre-implantation genetic diagnosis to select compatible embryo for cord blood transplantation as treatment for sickle cell anemia

**DOI:** 10.5935/1518-0557.20180017

**Published:** 2018

**Authors:** Ciro D Martinhago, Kalina RN Endo, Mariana A Oliveira, Alex MM Dias, Gislaine S Pereira, Augusto M Azzolini, Paula RQ Estrada, Caio G Bruzaca, Ana Carolina N Martinhago

**Affiliations:** 1 Chromosome Medicina Genômica, São Paulo, SP, Brazil

**Keywords:** sickle cell anemia, preimplantation genetic diagnosis, stem cell transplantation

## Abstract

Sickle cell anemia is an inherited systemic hemoglobinopathy that affects hemoglobin production in red blood cells, leading to early morbidity and mortality. It is caused by a homozygous nucleotide substitution (c.20A>T) in the β-globin gene (*HBB*) that changes a glutamic acid to a valine in the protein. We present a case report of a fertile couple, both carriers of the sickle cell anemia mutation, with one affected daughter. Six cycles of assisted reproductive techniques were performed, resulting in 53 embryos in cleavage stage. Each embryo was biopsied and analyzed for pre-implantation genetic diagnosis (PGD) by fluorescent polymerase chain reaction, using polymorphic markers of the region of interest followed by capillary electrophoresis in an automated genetic analyzer. HLA Compatible and normal embryos for the mutation represented 3 (5.66%); while the carriers and compatible 6 (11.32%); therefore, embryos matching those of the affected daughter represented 9 (16.98%). A selected embryo in blastocyst stage was transferred, resulting in a healthy male newborn, who had the umbilical cord blood cells collected and stored. The affected daughter was immunosuppressed and received transplanted cells from the umbilical cord blood of her brother; the treatment was successful. Embryo selection using PGD technologies represent the most effective treatment plan for parents who want to have a healthy child, and it could cure another child already affected by inherited hemoglobinopathy.

## INTRODUTION

Sickle cell anemia (MIM#603903) is an inherited hemoglobinopathy, caused by a homozygous nucleotide substitution (c.20A>T) in the β-globin gene (*HBB*) that changes a glutamic acid to a valine in the protein. It is an inherited hemoglobinopathy, such as β-thalassemia, and leads to a multisystem disorder characterized by abnormal erythrocytes damaged by the HbS hemoglobin, an adult hemoglobin (HbA) variant (Piel *et al*., 2017).

The HbS polymerizes, damaging the erythrocyte, leading to the formation of the sickle cells. Such as a multisystem disorder, SCA leads to a high morbimortality due to vaso-occlusion phenomena in small blood vessels that cause ischemic features, resulting in severe pain or even organ failure, as an acute complication (Piel *et al*., 2017).

The multidisciplinary treatment of SCA includes using drugs like hydroxyurea or blood transfusions, both palliatives. Hematopoietic stem cell transplant is an alternative to cure an individual affected by SCA; nevertheless, bone marrow transplantation needs a haploidentical donor to qualify a patient to this treatment, which is rare to find in bone marrow donor lists (Fernandes, 2017; Kassim & Sharma, 2017).

Using assisted reproductive techniques (ART's) and pre-implantation genetics diagnosis (PGD), it is now possible for a couple, both parents being carriers of the SC trait to have a child without SCA. It is also possible to genotype markers of the human leukocyte antigen region to select an embryo that will be a haploidentical donor to cure the affected child. (Kuliev *et al*., 2005; Xu *et al*., 1999).

We present the first South American case report of using cord blood stem cell transplantation, after using pre-implantation genetic diagnosis to select healthy and haploidentical embryos from a fertile couple, both with sickle cell trait, parents of an affected child.

## CASE REPORT

**Figure 1:**
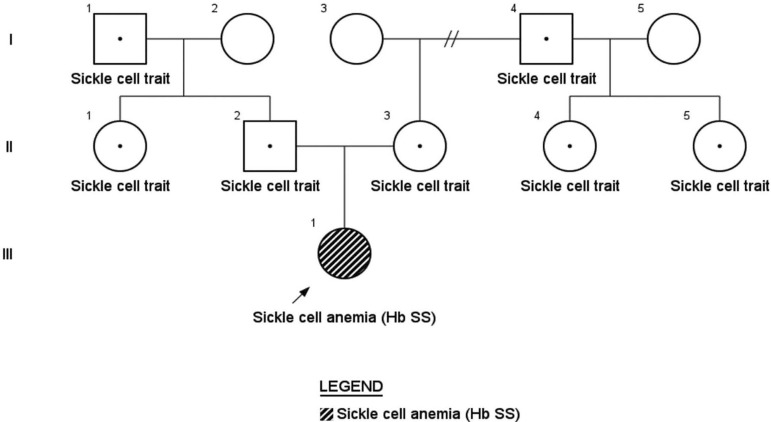
Pedigree of the family studied.

A fertile couple had an affected daughter with SCA. First, the parents underwent genetic counseling and it was decided to perform a linkage study of the *HBB* region. Blood was collected from the parents, the affected daughter and from other relatives that voluntarily agreed to be part of the genetic study (Figure 1). 

Genotyping was performed by fluorescent polymerase chain reaction (PCR) of short tandem repeat (STR) polymorphic markers located near the *HBB* gene, followed by capillary electrophoresis in an automated DNA genetic analyzer (ABI3500). After analyzing the PCR products, it was possible to identify which haplotype was segregating with the mutated alleles of the *HBB* gene from parents, and it was possible to know the genetic profile of markers of the human leukocyte antigen region.

**Figure 2:**
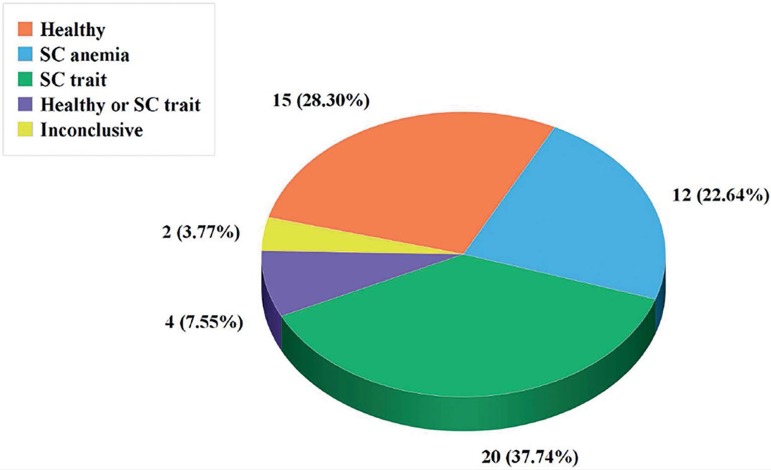
Evaluation of all embryos, according to SC anemia, trait or healthy (n=53).

**Figure 3:**
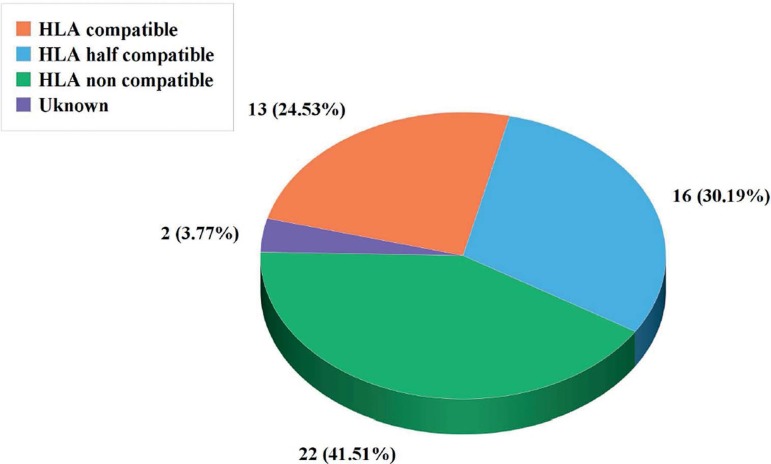
Evaluation of all embryos, according to HLA compatibility (n=53).

The couple underwent *in vitro* fertilization procedures that enabled the analysis of each embryo after biopsy. A total of 53 embryos were analyzed, of which 15 (28.3%) were normal for the *HBB* gene; 12 embryos (22.64%) were homozygous for the SCA mutation; 20 embryos were carriers with only one copy of the SCA mutation. For 4 embryos It was not possible to determine whether they were normal or carriers (7.55% of the cases) due to allelic loss; and for 2 embryos (3.77%), the results were inconclusive. For the HLA matching analysis, 13 embryos (24.53%) were compatible; 16 embryos (30.19%) were half-compatible and 22 embryos (41.51%) were not compatible (Figures 2 and 3).

**Figure 4:**
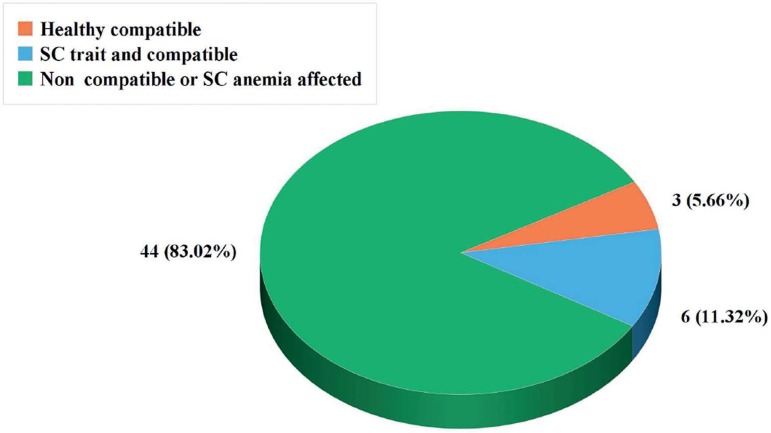
Evaluation of all embryos, according to SC and HLA compatibility (n=53).

A total of 9 embryos (16.98%) were HLA compatible with the affected daughter, three of them (5.66%) were normal for the SCA mutation and the other six embryos (11.32%) were carriers of the SCA mutation. The others 44 embryos (83.02%) were SCA affected and/or HLA incompatible (Figure 4).

A selected HLA compatible embryo, not homozygous for SCA mutation, in the blastocyst stage was transferred, resulting in a healthy newborn. The umbilical cord blood was collected and stored. The affected daughter was immunosuppressed and stem cells from the cord blood were transplanted; the treatment of the index case was successful.

## DISCUSSION

Hemoglobinopathies are the conditions for what PGD is utilized the most, but it has been reported for other diseases such as cystic fibrosis, beta thalassemia, Huntington disease and others. 

 In hemoglobinopathies, such as SC anemia and β-thalassemia, with the use of hematopoietic stem cell transplantation, life expectancy and quality of an affected can be incredible improved. Nevertheless, having an anonymous HLA compatible matching in bone marrow and cord banks is rare and difficult to find. Those conditions are well known to enable PGD use to select healthy and HLA haploidentical embryos (Kuliev *et al.*, 2011).

Selecting embryos with PGD technologies, parents with increased likelihoods of having a child with any monogenic disease, could yield an unaffected child. Clinical genetics and ART may now enable embryo selection to exclude a mutated gene from a family, such as in sickle cell anemia. For this disease, PGD can also select an HLA compatible embryo to be a stem cell donor for the affected child (Kuliev *et al*., 2005). The cord blood cells of the healthy child can be used to restore the hematopoietic bone marrow of the affected child (Kahraman *et al*., 2014). 

## CONCLUSION

PGD in hemoglobinopathies with HLA matching is a useful technique, and it provides a realistic option for couples seeking a healthy child and a treatment for an affected child when no compatible bone marrow donor is available.

## ABBREVIATIONS

ART: Assisted Reproductive Technology

PGD: Preimplantation Genetics Diagnosis

HLA: Human Leucocyte Antigen

SCA: Sickle Cell Anemia
